# Sotagliflozin, a dual SGLT1 and SGLT2 inhibitor: In the heart of the problem

**DOI:** 10.1016/j.metop.2021.100089

**Published:** 2021-03-24

**Authors:** Natalia G. Vallianou, Gerasimos Socrates Christodoulatos, Dimitris Kounatidis, Maria Dalamaga

**Affiliations:** First Department of Internal Medicine, Evangelismos General Hospital, 45-47 Ipsilantou Str, 10676, Athens, Greece; Department of Biological Chemistry, Medical School, National and Kapodistrian University of Athens, 75 Mikras Asias Str, Goudi, 11527, Athens, Greece; First Department of Internal Medicine, Evangelismos General Hospital, 45-47 Ipsilantou Str, 10676, Athens, Greece; Department of Biological Chemistry, Medical School, National and Kapodistrian University of Athens, 75 Mikras Asias Str, Goudi, 11527, Athens, Greece

**Keywords:** Cardiovascular disease, Diabetes, Heart failure, Sodium glucose co-transporters inhibitor, Sotagliflozin

During the Covid-19 pandemic, we can lose sight of the fact that diabetes is also a pandemic, affecting 400 million individuals and causing approximately more than 1.3 million deaths in 2016, a burden of mortality similar to what has been reported from Covid-19 in one year [1,2]. Sodium Glucose Co-transporters 2 (SGLT2) inhibitors are licensed for the treatment of type 2 diabetes mellitus (T2DM), and more recently for heart failure with or without diabetes. Evidence from Randomized Controlled Trials (RCTs) has demonstrated that SGLT2 inhibitors show notable antihyperglycemic efficacy combined with an adequate cardiovascular (CV) safety profile, or even CV event reduction is some cases [3]. SGLT2 inhibitors provide impressive cardiac and renal outcome benefits to subjects with and without T2DM [4].

Sotagliflozin is a dual inhibitor of SGLT2 and SGLT1, the former dominating in the kidney and the latter in the small intestine [5]. SGLT2 transporters are mainly located in the proxy part of the renal proximal tubule and are responsible for the re-absorption of approximately 90% of the filtered glucose. SGLT2 transporters are expressed in the kidneys, the brain, the heart, the liver, the thyroid gland and the muscles. SGLT-2 inhibitors impede renal glucose re-absorption and act by causing glucosuria.

**Fig. 1:**
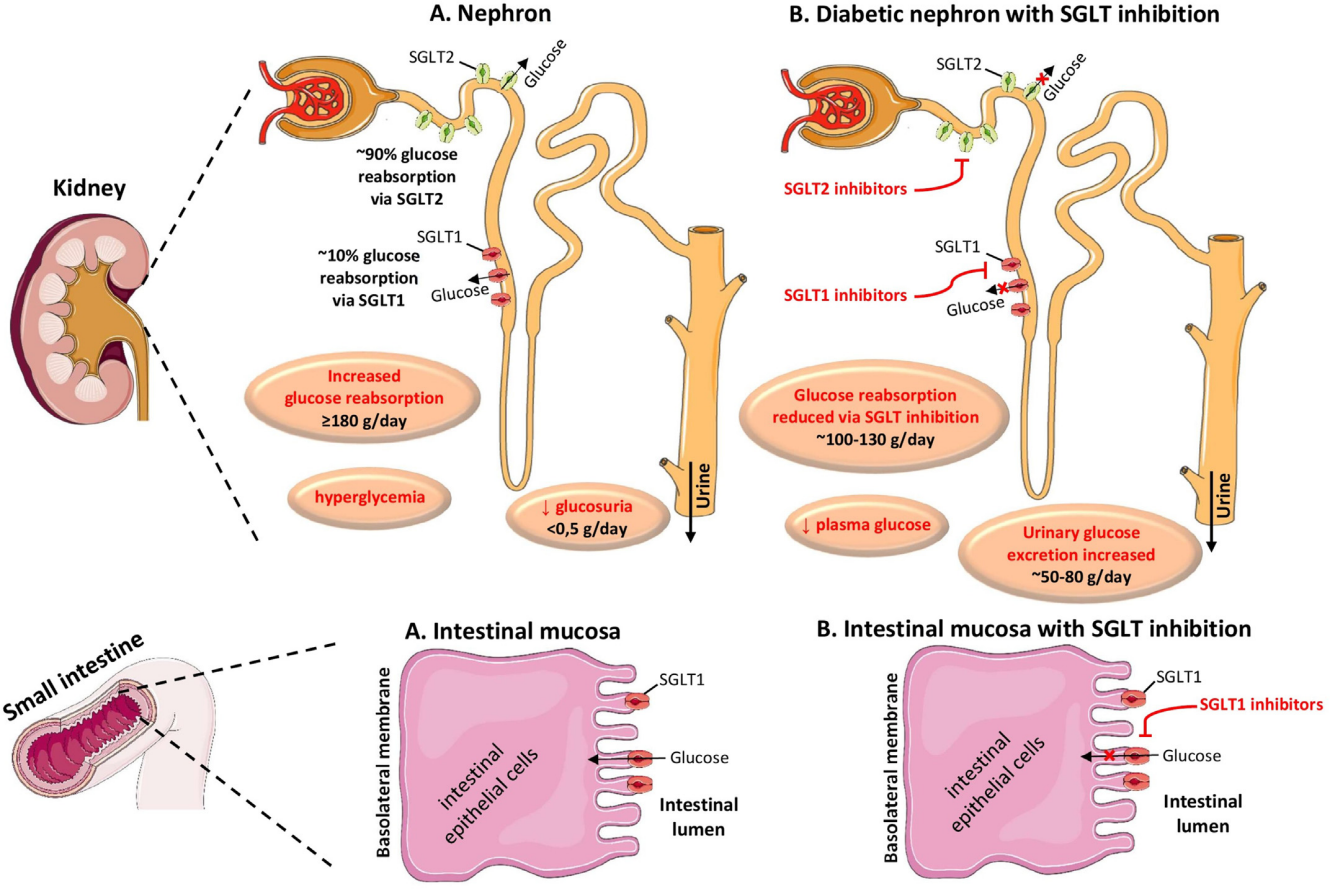
SGLTs; Sodium Glucose Co-transporters. SGLT1 and SGLT2 are proteins found in the intestinal mucosa of the small intestine (SGLT-1) and the proximal tubule of the nephron (SGLT1 and SGLT2) playing an important role in maintaining blood glucose balance. In physiologic conditions, SGLT1 is responsible for glucose absorption in the small intestine, and for the reabsorption of nearly 10% of the filtered glucose load, while SGLT2 is responsible for approximately 90% of the kidney’s glucose reabsorption. The selective SGLT2 inhibitors empagliflozin, canagliflozin, dapagliflozin and ertugliflozin act by inhibiting SGLT2 in the kidney, while the dual SGLT1 and SGLT2 inhibitor sotagliflozin acts by inhibiting both renal SGLT2 and intestinal SGLT1. (All images are derived from the free medical site http://smart.servier.com/by Servier licensed under a Creative Commons Attribution 3.0 Unported License).

SGLT1 transporters are located in the intestines, the kidney, the brain, the heart, the trachea, the testis and the prostate gland [6,7]. SGLT1 transporters are responsible for glucose absorption in the small intestine, and for the re-absorption of approximately 10% of the filtered glucose in the upper part of the renal proximal tubule (Fig. 1). SGLT1 inhibitors act by delaying glucose absorption in the small intestine mainly, thus resulting in a decrease in the serum postprandial glucose levels [6,7].

Apart from controlling blood glucose levels, gliflozins have been shown to provide significant CV benefit in T2DM patients [3,4]. Several medications of this class have been approved or are currently under development. There are now four selective SGLT2 inhibitors with demonstrated cardiovascular benefit, i.e. empagliflozin, canagliflozin, dapagliflozin, and ertugliflozin [8]. The abovementioned drugs have been documented to improve blood glucose control, decrease body weight and reduce systolic and diastolic blood pressure as well, probably through osmotic diuresis (glucosuria), and natriuresis [8]. SGLT2 Inhibitors and their characteristics are depicted in Table 1.

**Table 1:** List of SGLT2 Inhibitors and their characteristics.

Gliflozin	Approvals	Bioavailability (%)	Selectivity of SGLT2 over SGLT1 (fold)	Dose (mg)	Remarks	Adverse effects
Empagliflozin	FDA in 2014, EMA in 2014	78	2500	10/25	All SGLT2 Inhibitors should be administered with caution, especially in the elderly patients who have been receiving diuretics, as the risk of DKA is increased due to the glucosuria and the osmotic diuresis that they induce.	Genitourinary Infections (mostly candiduria) Euglycemic DKA Fournier’s Gangrene
Canagliflozin	FDA in 2013, EMA in 2013	65	200	100/300	The increased risk for amputations has been demonstrated only with the use of canagliflozin and it is not yet known whether it represents a class effect or a specific drug effect.	Genitourinary Infections (mostly candiduria) Euglycemic DKA Fournier’s Gangrene ↑ Risk of lower limb amputation
Dapagliflozin	FDA in 2014, EMA in 2012	78	1200	5/10	DKA is usually associated with only slightly abnormal levels of serum glucose levels, i.e. it is a euglycemic DKA, which is characteristic of this class of drugs.	Genitourinary Infections (mostly candiduria) Euglycemic DKA Fournier’s Gangrene
Ertugliflozin	FDA in 2017, EMA in 2018	70–90	2000	15	Patients need to be careful regarding their hydration status in order to avoid this highly preventable adverse effect.	Genitourinary Infections (mostly candiduria) Euglycemic DKA Fournier’s Gangrene
Ipragliflozin	Japan in 2014	90	360	50	This class of drugs should be stopped 3 days before surgery to decrease the risk of DKA.	Genitourinary Infections (mostly candiduria) Euglycemic DKA Fournier’s Gangrene
Luseogliflozin	Japan in 2014		1650	2.5/5		Genitourinary Infections (mostly candiduria) Euglycemic DKA Fournier’s Gangrene
Tofogliflozin	Japan in 2014	97.5	2900	10		Genitourinary Infections (mostly candiduria) Euglycemic DKA Fournier’s Gangrene

Abbreviations: DKA: Diabetic ketoacidosis; EMA: European Medicinal Agency; FDA: Food and Drug Administration; SGLTs; Sodium Glucose Co-transporters.

Only recently, Bhatt et al. have reported the results of two RCTs on the CV and renal effects of sotagliflozin. The first trial, Sotagliflozin in Patients with Diabetes and Recent Worsening Heart Failure (SOLOIST-WHF), enrolled 1222 participants with T2DM, with a recent episode of decompensated heart failure (HF), in order to determine the efficacy and safety of sotagliflozin in preventing future HF events or CV death [9]. The second study, Effect of Sotagliflozin on Cardiovascular and Renal Events in Patients with Type 2 Diabetes and Moderate Renal Impairment (SCORED), enrolled 10,584 patients in order to determine whether sotagliflozin could reduce the total number of deaths from CV causes in conjunction with hospitalizations for HF among patients with T2DM and chronic kidney disease (CKD), regardless of the degree of albuminuria [10].

In the SOLOIST-WHF trial, sotagliflozin therapy initiated before or shortly after discharge in patients with T2DM and recent worsening HF, led to a significantly lower total number of deaths from CV causes and hospitalizations, and urgent visits for HF, which was the primary composite outcome of the study, than placebo [9]. The rate of death (the number of deaths per 100 patient-years) from CV causes was 10.6 in the sotagliflozin group and 12.5 in the placebo group (hazard ratio [HR]: 0.84, 95% CI: 0.58–1.22), while the rate of death from any cause was 13.3 in the sotagliflozin group and 16.3 in the placebo group (HR: 0.82, 95%CI: 0.59–1.14). Diarrhea and severe hypoglycemia were more frequent with sotagliflozin than with placebo (6.1% vs. 3.4% and 1.5% vs. 0.3% respectively). In the SCORED trial, for the end point of the first occurrence of death from CV causes, non-fatal myocardial infarction and non-fatal stroke, the HR was 0.84 (95% CI: 0.72–0.99), while the HR for the first occurrence of death from CV causes or hospitalization due to HF was 0.77 (95% CI: 0.66–0.91). However, diarrhea, volume depletion, genital mycotic infections, and diabetic ketoacidosis were more frequent with sotagliflozin than with placebo [10].

It is noteworthy that both trials were stopped early due to the loss of funding from the sponsor, and subsequently their original primary composite end points were defined again in order to gain statistical power. Nevertheless, this dual SGLT inhibitor performed almost equally well as the current selective SGLT2 inhibitors have performed until today, in terms of CV deaths and hospitalizations for HF [8].

In particular, among more than 280,000 patients, who have been included in the major trials with selective SGLT2 inhibitors, significant reductions in the relative risks of individual adverse CV and kidney events, such as progression to end stage renal disease (ESRD), progression to macroalbuminuria and a two-fold increase in serum creatinine levels were reported, despite an increased incidence of urogenital infections, especially urogenital candidiasis. Notably, the frequency of diabetic ketoacidosis (DKA) associated with sotagliflozin in the SCORED trial was almost the same as that found in the former CV studies with selective SGLT2 inhibitors. Therefore, the addition of intestinal SGLT1 inhibition appeared to affect neither the beneficial effects nor the potential harms related to the use of sotagliflozin. Therefore, it seems likely that sotagliflozin will be added to the list of recommended SGLT2 inhibitors for T2DM. Sotagliflozin is authorized in the European Union only for treatment of patients with both T1DM and obesity [8]. As less than one third of patients with T1DM achieve glycemic goals and most are overweight or obese, sotagliflozin is indicated in the European Union for patients with both T1DM and obesity, offering a better glycemic control by reducing postprandial hyperglycemia through its action in SGLT1 in conjunction with its anti-obesogenic potential, which is attributed mainly to its glucosuric effects in the kidney [4,11]. Sotagliflozin is not currently approved for any indication in the United States, but this is very likely to change in the near future, based on the results by Bhatt et al. [9,10].

However, many questions arise on how these cardiac beneficial effects of SGLT inhibitors could be explained. In a 2015 study, Vrhovac et al. has used purified antibodies for human SGLT1 (hSGLT1) and SGLT2 (hSGLT2), and thereby identified locations of SGLT2 in the human kidney and SGLT1 in the human kidney, small intestine, liver, lung and heart [12]. SGLT1 inhibition in the heart may be an additional mechanism of cardioprotection.

Mitochondria are known to sequester huge amounts of Ca^++,^ which is a key regulator of energy production as well as mitochondrial morphology and apoptosis [12]. In the Zucker diabetic fatty/spontaneously hypertensive heart failure (ZSF) animal model of HF with preserved ejection fraction (HfpEF), an increased mitochondrial Ca^++^of cardiomyocytes in the left ventricle at rest has been related to an elevated increased cytosolic Ca^++^, mitochondrial swelling and reduced mitochondrial respiration [11]. Bode et al. have documented that dual SGLT1 and SGLT2 inhibition with sotagliflozin resulted in normalization of the abnormal mitochondrial swelling of cardiomyocytes in the left atrium in HFpEF and enhanced mitochondrial Ca^++^ buffer capacity. A reduced cytosolic concentration of Na^+^ or Ca^++^ at rest could account for this effect [13]. Mitochondrial Ca^++^ uptake has been demonstrated to contribute to the buffering of cytosolic Ca^++^ peaks in cardiomyocytes, whilst pharmacologic enhancement of mitochondrial Ca^++^ uptake has been related to a decreased sarcoplasmic reticulum concentration of Ca^++^ in catecholaminergic ventricular tachycardia models. An enhanced mitochondrial Ca^++^ buffer capacity has been suggested to contribute to decreased sarcoplasmic reticulum Ca^++^ levels and less ventricular arrhythmias. Moreover, mitochondrial swelling has been attributed to a Ca^++^ overload, thus leading to an opening of the mitochondrial permeability transition pore, mitochondrial depolarization, generation of reactive oxygen species and subsequent apoptosis [11,12]. Bode et al. have additionally reported an improvement in left atrium remodelling in their experiment in rat models. This increased buffer capacity of mitochondrial Ca^++^ may account for the subsequent decrease in the size of the cardiomyocytes’ mitochondria by means of reversing mitochondrial swelling [13].

Moreover, sotagliflozin has also been demonstrated to provide beneficial effects on blood pressure and body weight, partially due to the glucosuria as well as the increase in the secretion of glucagon like peptide-1 (GLP-1) due to the delayed glucose intestinal absorption [12,13].

In conclusion, sotagliflozin has only recently been documented to prevent deaths from CV causes and hospitalizations and urgent visits for HF among patients with T2DM and recent worsening of HF, as well as among patients with T2DM and CKD, with or without albuminuria. Nevertheless, further large-scale studies are needed to confirm the molecular mechanisms behind the cardiac beneficial effects of dual SGLT inhibition. It is high time to focus on the heart of the problem. At last, it may be time to apply this dual SGLT inhibitor in our armamentarium against T1DM and T2DM, especially among patients with HF and CKD.
